# Inhibition of Granulomatous Inflammation and Prophylactic Treatment of Schistosomiasis with a Combination of Edelfosine and Praziquantel

**DOI:** 10.1371/journal.pntd.0003893

**Published:** 2015-07-20

**Authors:** Edward Yepes, Rubén E. Varela-M, Julio López-Abán, Jose Rojas-Caraballo, Antonio Muro, Faustino Mollinedo

**Affiliations:** 1 IBSAL-CIETUS (Instituto de Investigación Biomédica de Salamanca-Centro de Investigación de Enfermedades Tropicales de la Universidad de Salamanca), Facultad de Farmacia, Universidad de Salamanca, Salamanca, Spain; 2 Instituto de Biología Molecular y Celular del Cáncer, Centro de Investigación del Cáncer, CSIC-Universidad de Salamanca, Campus Miguel de Unamuno, Salamanca, Spain; 3 Instituto de Investigación Biomédica de Salamanca (IBSAL), Hospital Universitario de Salamanca, Salamanca, Spain; Biomedical Research Institute, UNITED STATES

## Abstract

**Background:**

Schistosomiasis is the third most devastating tropical disease worldwide caused by blood flukes of the genus *Schistosoma*. This parasitic disease is due to immunologic reactions to *Schistosoma* eggs trapped in tissues. Egg-released antigens stimulate tissue-destructive inflammatory and granulomatous reactions, involving different immune cell populations, including T cells and granulocytes. Granulomas lead to collagen fibers deposition and fibrosis, resulting in organ damage. Praziquantel (PZQ) is the drug of choice for treating all species of schistosomes. However, PZQ kills only adult *Schistosoma* worms, not immature stages. The inability of PZQ to abort early infection or prevent re-infection, and the lack of prophylactic effect prompt the need for novel drugs and strategies for the prevention of schistosomiasis.

**Methodology/Principal Findings:**

Using *in vitro* and *in vivo* approaches, we have found that the alkylphospholipid analog edelfosine kills schistosomula, and displays anti-inflammatory activity. The combined treatment of PZQ and edelfosine during a few days before and after cercariae infection in a schistosomiasis mouse model, simulating a prophylactic treatment, led to seven major effects: a) killing of *Schistosoma* parasites at early and late development stages; b) reduction of hepatomegaly; c) granuloma size reduction; d) down-regulation of Th1, Th2 and Th17 responses at late post-infection times, thus inhibiting granuloma formation; e) upregulation of IL-10 at early post-infection times, thus potentiating anti-inflammatory actions; f) down-regulation of IL-10 at late post-infection times, thus favoring resistance to re-infection; g) reduction in the number of blood granulocytes in late post-infection times as compared to infected untreated animals.

**Conclusions/Significance:**

Taken together, these data suggest that the combined treatment of PZQ and edelfosine promotes a high decrease in granuloma formation, as well as in the cellular immune response that underlies granuloma development, with changes in the cytokine patterns, and may provide a promising and effective strategy for a prophylactic treatment of schistosomiasis.

## Author Summary

Schistosomiasis is one of the most devastating tropical diseases worldwide caused by blood flukes of the genus *Schistosoma*. Schistosomiasis results from immune-mediated granulomatous responses against *Schistosoma* eggs trapped in tissues, causing serious local and systemic pathological effects because of granuloma formation and fibrosis. Treatment and control of schistosomiasis relies almost entirely on the single drug praziquantel (PZQ). This drug kills adult *Schistosoma* worms, but has poor activity against immature worms, thus leading to low cure rates in schistosomiasis-endemic areas that could reflect infections through PZQ-refractory juvenile worms due to high rates of transmission. At present, there is a lack of an efficient prophylactic treatment for schistosomiasis that could be critical for highly endemic areas, as well as for travelers to these regions. Here, we have found that a prophylactic combination treatment of PZQ with the ether lipid edelfosine, which is able to kill schistosomula, promotes a significant decrease in granuloma development and in the inflammatory response underlying granuloma formation, thus leading to a promising prophylactic treatment of schistosomiasis. In addition, a high decrease in IL-10 and IL-17 levels following the combined prophylactic treatment of PZQ and edelfosine might potentiate inhibition of granuloma formation and resistance to *S*. *mansoni* re-infection.

## Introduction

Schistosomiasis is caused by blood flukes (trematodes) belonging to the genus *Schistosoma*. *Schistosoma* spp. parasites need two hosts for their survival, namely an intermediate snail host, where asexual reproduction takes place and a definitive mammalian host, where the sexual reproduction occurs [1, 2]. Schistosomiasis is the most important water-borne disease, being the main human helminth infection in terms of global mortality and the third most devastating tropical disease in the world, following malaria and intestinal helminthiasis, and causing both significant morbidity and mortality on several continents [3–7]. The bulk of morbidity due to schistosomiasis results from cellular immune responses and the generation of cytokine patterns, elicited during the different stages of the parasite’s life cycle in the course of infection, that eventually lead to chronic immune response-based inflammation against *Schistosoma* eggs lodged in tissues, and subsequent granuloma formation and fibrosis [8, 9]. Symptoms and signs of the disease depend on the number and location of eggs trapped in the tissues, leading first to a reversible inflammatory reaction and then to the pathology associated with collagen deposition and fibrosis, resulting in organ damage [9, 10]. Most human schistosomiasis is caused by *Schistosoma haematobium*, *S*. *mansoni*, and *S*. *japonicum* [6, 11–13]. The World Health Organization (WHO) estimates that schistosomiasis is endemic in 74 developing countries, infecting at least 230 million people in rural and peri-urban areas worldwide (80% in sub-Saharan Africa). Of these, ~120 million have symptoms of the disease, and ~20 million have severe disease, resulting in approximately 280,000 deaths annually [2, 4, 7, 14]. Human infection occurs by direct contact with *S*. *mansoni* cercariae-contaminated water. Following penetration of cercariae through the skin, they lose their tails and transform into schistosomula. The schistosomula then enter the venous system and reach the lungs, where they mature to pre-adult stages. About 8–10 days after infection, the pre-adult forms reach the portal system, where they mature to adult males and females [1, 5, 9]. Both male and female *S*. *mansoni* parasites achieve sexual maturity in the bloodstream, and then sexual reproduction occurs with the deposition of hundreds of eggs per day [12, 15, 16], predominantly in the liver and intestine. Deposition of schistosome eggs in the tissues is an important stimulus to the influx of immune cells that leads to the development of a granulomatous reaction. This immunological reaction protects the host by neutralizing the schistosome eggs antigens and destroying eggs. However, schistosome eggs elicit a CD4^+^ T-helper (Th) cell-mediated hepatic granulomatous inflammation, which is the major pathological consequence of the disease [15, 16]. Nevertheless, paradoxically, the development of granulomatous inflammation around parasite eggs has an essential host-protective and facilitates the successful excretion of the eggs from the host [14, 16, 17]. Two main clinical conditions are recognized in *S*. *mansoni*-infected individuals: acute schistosomiasis and chronic schistosomiasis. Acute schistosomiasis in humans is a debilitating febrile illness (Katayama fever) that can occur before the appearance of eggs in the stool and generally peaks around six to eight weeks after infection [18]. Cytokine production by peripheral blood mononuclear cells after stimulation with parasite antigen reflects a dominant T helper 1 (Th1) response, with production of interferon-γ (IFN-γ) and interleukin-2 (IL-2) [19]. Thus, during the acute phase of the disease there is a predominance of a Th1 response, producing elevated levels of Th1 cytokines in the plasma [17]. Then, in the natural progression of the disease, after parasites mature, mate and start to produce eggs at the fifth-sixth week, the initial Th1 response is followed by a developing egg antigen-induced regulatory T cell (Treg cell) and T helper 2 (Th2) response that downregulates the production and effector functions of the pro-inflammatory Th1 mediators with accompanying granuloma formation [15, 20, 21]. Treg and Th2 cells share some features, notably their ability to synthesize interleukin-10 (IL-10) through which suppress the development of Th1 responses to schistosome egg antigens, thus cooperating both cell types to enforce the Th2 polarization that characterizes the immune response in schistosome-infected mice [22]. The production of IL-10 during this latter period seems to have an important role in hepatic granuloma formation and in the regulation of CD4^+^ T cell responses in schistosomiasis, as well as in the transition from acute to chronic disease state [17, 23–25]. In the mouse model, both Th1 and Th2 cytokines can orchestrate granuloma development [16, 25, 26]. Th2–type responses are typically characterized by increases in the levels of interleukin-4 (IL-4) and other cytokines (including IL-5, IL-6, IL-9, and IL-13), activation and expansion of CD4^+^ Th2 cells, plasma cells secreting IgE, eosinophils, mast cells and basophils [16, 27]. IL-17 is the signature cytokine of the proinflammatory Th17 cell population [28, 29], and a subsequent Th17 response is elicited during infection that plays a major role for full deployment of inflammation [30] and for the development of severe schistosome egg-induced immunopathology [31]. Elucidation of the actual determinants of immunomodulation in human or murine schistosomiasis could lead to the development of drugs or vaccines for disease control or to spin-off benefits for other granulomatous diseases [16]. Praziquantel (PZQ) is currently the only available antischistosomal drug and it is distributed through mass administration programs to millions of people every year, thus increasing the risk for drug resistance, and therefore search for new antischistosomal drugs and therapeutic approaches is urgently needed [7, 32]. Adult worms are highly sensitive to PZQ, but unfortunately this drug has minor activity against juvenile stages like schistosomula, pre-adults and juvenile adults [32]. Despite the paucity of a concerted effort to develop novel antischistosomal drugs, with a lack of dedicated drug discovery and development programs pursued for schistosomiasis, a number of compounds with promising antischistosomal properties have been recently identified, such as the alkylphospholipid analogs (APLs) [33–35]. APLs are a class of structurally related synthetic lipid compounds, including edelfosine (EDLF) and miltefosine, which act on cell membranes rather than on DNA [36, 37]. EDLF (1-*O*-octadecyl-2-*O*-methyl-*rac*-glycero-3-phosphocholine), considered as the prototype APL molecule, is a promising antitumor ether phospholipid drug [38, 39], that acts by activating apoptosis through its interaction with cell membrane domains [36, 37, 40–42]. Interestingly, the APL miltefosine is currently being used in the clinic for the treatment of human and animal leishmaniasis [43, 44], and the APL EDLF has been reported to display anti-inflammatory properties [45] and to modulate cytokine production, including IFN-γ, IL-2 and IL-10 [45–47]. EDLF has also been shown to cause interruption of oviposition in a preliminary *in vitro* screening, and a significant reduction in worm burden *in vivo*, with a preferential activity against male worms [35]. Here, using both *in vitro* and *in vivo* approaches, we have found that EDLF is able to kill juvenile stages as schistosomula, and the combination of PZQ and EDLF behaves as a promising prophylactic treatment against schistosomiasis, showing a significant reduction in adult worm burden, number of parasite eggs in liver and intestine tissues and granuloma size, as well as exerting an anti-inflammatory action, through modulation of cytokine production in infected mice, that might be of special importance for the treatment and/or prevention of schistosomiasis.

## Materials and Methods

### Ethics statement

Animal procedures in this study complied with the Spanish (Ley 32/2007, Ley 6/2013 and Real Decreto 53/2013) and the European Union (European Directive 2010/63/EU) regulations on animal experimentation for the protection and humane use of laboratory animals, and were conducted at the accredited Animal Experimentation Facility of the University of Salamanca (Register number: PAE/SA/001). Procedures were approved by the Ethics Committee of the University of Salamanca (protocol approval number 48531). The animals’ health status was monitored throughout the experiments by a health surveillance program according to Federation of European Laboratory Animal Science Associations (FELASA) guidelines. All efforts were made to minimize suffering.

### Drugs

EDLF was obtained from R. Berchtold (Biochemisches Labor, Bern, Switzerland). Stock sterile solutions of EDLF (2 mM) were prepared in culture medium by heating at 50°C for 30 min as previously described [38]. PZQ was obtained as Biltricide tablets (Bayer Vital, Leverkusen, Germany) and was dispersed in water with 2–2.5% Cremophor A6 oil-in-water emulsifier (Sigma, MO).

### Parasite culture and maintenance

*S*. *mansoni* (LE strain) was maintained by passage through *Biomphalaria glabrata* snails and 4- to 6-week-old male SPF (Specific Pathogen Free) Swiss CD1 mice from Charles River laboratory—CRIFFA (Barcelona, Spain). Mice were infected with abdominal percutaneous exposure to 150 *S*. *mansoni* cercariae per animal. Eight weeks after infection mice were humanely euthanized by intraperitoneal injection of sodium pentobarbital (60 mg/kg) plus heparin (2 IU/mL). The liver was removed and minced to obtain eggs to be hatched for harvesting miracidia and subsequent infection of snails. Cercariae were shed from infected snails by exposure to light (60 min at room temperature), and mechanically transformed into schistosomula by passing back and forth the parasites between two 10-mL syringes joined by a 22-gauge double-ended luer lock needle [48]. Schistosomula were purified away from cercarial tails by centrifugation through a 60% Percoll gradient as described previously [48]. Schistosomula were washed thrice in RPMI-1640 culture medium (Invitrogen, Carlsbad, CA), kept at pH 7.5 with 20 mM HEPES, and supplemented with antibiotic/antimycotic, as previously described [48], and then transferred to modified Basch’s medium at 37°C in an atmosphere of 5% CO_2_ for 24 h before any further experimental manipulations proceeded (S1 Video) [49, 50].

### *In vitro* schistosomula viability assay

The principle of this assay is based on the differential membrane permeability to the membrane-impermeable fluorescent DNA intercalating agent propidium iodide, staining membrane-compromised cells (red fluorescence) [51]. After 24 h of culturing (37°C, 5% CO_2_) in the presence of 10 and 20 μM EDLF, schistosomula were washed thrice to remove the test compound and culture media supplements. Each wash consisted of centrifuging microtiter plates containing schistosomula at 100 x *g* for 5 min, removal of half the old culture media and replacement with an equal amount of fresh Dulbecco’s Modified Eagle Medium (DMEM) (lacking phenol red). After washing the parasites, propidium iodide (2.0 μg/mL, final concentration) was simultaneously added to each well of the microtiter plate. The 96-well microtiter plates (containing ~100 parasites/well in triplicate), were subsequently loaded into a BioTek Synergy 2 plate reader (BioTek Instruments, Winooski, VT) containing appropriate filters for propidium iodide detection (485/20 excitation, 645/20 nm emission). The plate reader automatically sets the photo multiplier tube gain for the fluorescent dye and this may slightly vary between experiments. Inclusion of appropriate control samples (live and heat-killed dead schistosomula) compensates for any inter-plate variations in gain settings. Propidium iodide stains dead schistosomula, and then fluorescent intensity is determined to assess schistosomula viability, which could be quantified using a plate reader. A higher value of relative fluorescence units (RFU) indicates a higher number of dead parasites. Percentage of dead schistosomula was calculated using the following equation previously used by Peak *et al*. [51]: % of dead schistosomula = (sample - media control/negative control - media control) x 100, where “sample” represents RFU values from parasites treated with EDLF; “negative control” represents RFU values from parasites killed with heat shock (10 min incubation at 56°C); and “media control” represents RFU values from wells containing only medium (no parasites).

In addition, schistosomula parasite death was also assessed under optical microscope by morphologic changes (granular appearance and tegument defects) and loss of motility. S1 Fig shows the differential morphology and propidium iodide permeability between live and dead parasites.

### *In vivo* studies for testing the efficacy of EDLF and PZQ treatments

**Fig 1:**
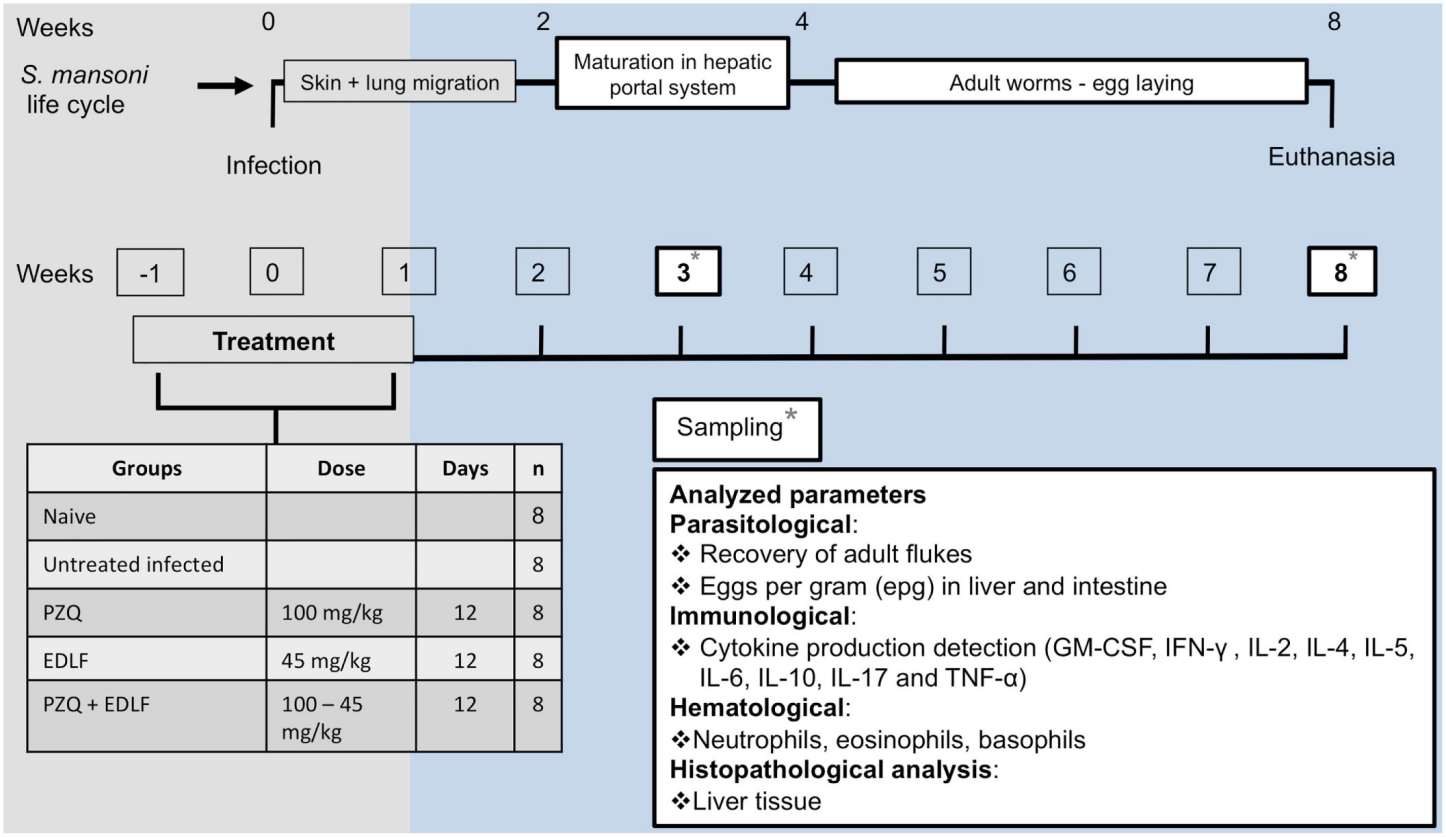
Experimental design for *in vivo* experiments. This scheme depicts schematically the studies conducted with *S*. *mansoni*-infected mice (n = 8) in this present work. Mice were treated daily (oral administration) with PZQ, EDLF or PZQ+EDLF since three days before animals were infected until eight days after infection. The untreated infected control group received only the vehicle solution used for 12 days. Animals that were untreated and uninfected (naive) were also run in parallel. Asterisks indicate when samples from animals were taken (sampling) to analyze the parameters indicated in the box. Animals were sacrificed at 8 weeks p.i., and the timeline of some major events in parasite life cycle and disease-related processes are indicated at the top of the scheme. See text for further details.

A total of forty 6-week-old female SPF Swiss CD1 mice from Charles River laboratory Spain (CRIFFA S.A., Barcelona), weighing 16–25 g, were infected by abdominal percutaneous exposure to 150 *S*. *mansoni* cercariae per animal [48], and randomly allocated into five experimental groups (8 animals per group) as follows: naive, untreated and uninfected; infected untreated; PZQ, treated with PZQ and infected; EDLF, treated with EDLF and infected; PZQ+EDLF, treated with PZQ + EDLF and infected. Mice were treated daily, since three days before animals were infected until eight days after infection, with PZQ (100 mg/kg/day), EDLF (45 mg/kg/day) and the combination PZQ+EDLF with the same individual drug doses, orally administered. The experimental design followed is shown in Fig 1. The infected untreated control group received only the vehicle solution used for 12 days.

Animals were humanely euthanized at 8 weeks post-infection (p.i.), and the following parasitological parameters were assessed: (i) worm burden through the recovery of parasites from hepatic and portomesenteric veins by using the Smithers and Terry perfusion technique for mice [48]; (ii) number of eggs per gram (epg) of hepatic and intestine tissues, by weighing fragments (about 0.3 g) of these tissues and subsequent processing by using the potassium hydroxide (KOH) digestion technique [52]; (iii) number of granulomas on liver. In addition, liver and intestine of each animal were harvested and adult worms were collected and counted. Portions of livers were collected for histological examination. Relative liver weight was calculated using the following equation [53]: relative liver weight = (absolute liver weight/body weight) x 100. Blood samples were taken at the beginning of the study, at the third week p.i., and after 8 weeks p.i. when animals were killed.

### Histopathological analysis

After killing the mice at week 8 p.i., liver sections were removed from the central part of the left lateral lobe and fixed in 4% formalin. Histological section were cut using a microtome at a thickness of 4 μm and stained on a slide with hematoxylin and eosin [54–56]. The slides were viewed using an Olympus BX51 microscope (Olympus, Center Valley, PA). Images were captured using a DP70 digital camera and the DP Controller software (Olympus). Granuloma diameters (five granulomas per mouse) were measured in a horizontal plane bisecting central eggs [57, 58] using the Olympus DP Controller software.

### Hematological techniques

Blood samples were collected in vacutainer tubes, containing EDTA as anticoagulant, with gentle shaking. Total white blood cells were quantified using a Hemavet HV950 system (Drew Scientific Co. Limited, Barrow in Furness, UK).

### Cytokine determination in mouse sera samples

A flow cytometry-based technique was used for cytokine quantitation (IFN-γ, GM-CSF, IL-2, IL-4, IL-5, IL-6, IL-10 and IL-17) from mice sera. A FlowCytomix Mouse Th1/Th2 kit (Bender MedSystems GmbH, Vienna, Austria) was used according to the manufacturer’s instructions. Briefly, different sized fluorescent beads, coated with capture antibodies specific for the aforementioned cytokines were incubated with mouse sera samples and with biotin-conjugated secondary antibodies for 2 h at room temperature. The specific antibodies bind to the analytes captured by the first antibodies. After washing the tubes with PBS plus 2% fetal calf serum, Streptavidin-Phycoerythrine (S-PE) solution was added and incubated at room temperature for 1 h. S-PE binds to the biotin conjugate and emits fluorescent signals. Flow cytometry data were collected using a FACSCalibur flow cytometer (BD Biosciences) (8000 events were collected, gated by forward and side scatter), and data were analyzed using FlowCytomix Pro 3.0 software (Bender MedSystems, Vienna, Austria). Each cytokine concentration was determined from standard curves using known mouse recombinant cytokine concentrations.

### Statistical analysis

Results were analyzed in GraphPad Prism Version 5 (Graphpad Software Inc.) and expressed as means ± SEM. Test for normality was performed by Kolmogorov-Smirnov, and then one-way ANOVA analyses of variance, followed by Dunnett’s or Kruskall Wallis comparison test, were performed to determine any statistical differences between treated groups and untreated controls. Data were considered significant if *p*-value was < 0.05.

## Results

### *In vitro* schistosomula viability determination in response to EDLF

**Fig 2:**
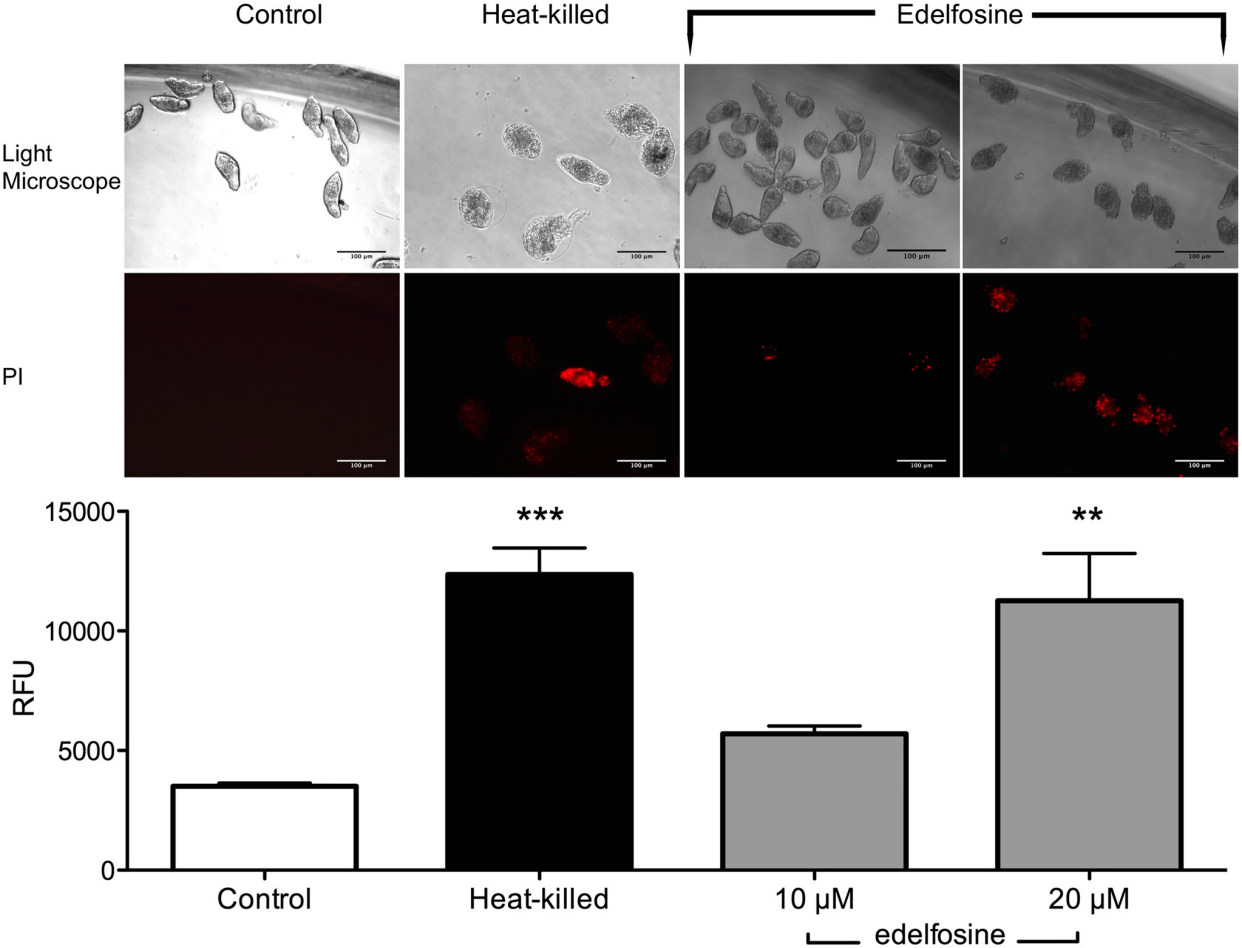
*In vitro* effects of EDLF on the viability of *S*. *mansoni* schistosomula. Schistosomula were untreated (Control), heat-killed at 56°C, or treated with 10 or 20 μM EDLF for 24 h. Then, schistosomula viability was analyzed by propidium iodide (PI) incorporation and light microscopy morphology as shown in Materials and Methods. RFU, relative fluorescence units. Data are shown as means ± SEM of three separate experiments. Asterisks represent statistical significance with respect to control-live group. **, *p*<0.01; ***, *p*<0.001. Scale bar, 100 μm.

Because propidium iodide is not permeable to viable cells, PI incorporation could be used as a means of parasite killing. We found that schistosomula treated with 20 μM EDLF were stained with propidium iodide at a similar level as that of heat-killed parasites used as a positive killing control (Fig 2). Edelfosine induced schistosomula death as assessed by propidium iodide staining and morphological changes under microscopic observation (Fig 2 and S2 Video). Quantification of dead parasites, following the above two approaches, as indicated in the Materials and Methods section, showed that about 91% of schistosomula were killed upon 20 μM EDLF treatment for 24 h.

### Combined prophylactic treatment of PZQ and EDLF reduces adult worm count and decreases the size of hepatic granulomas in a schistosomiasis mouse model

**Fig 3:**
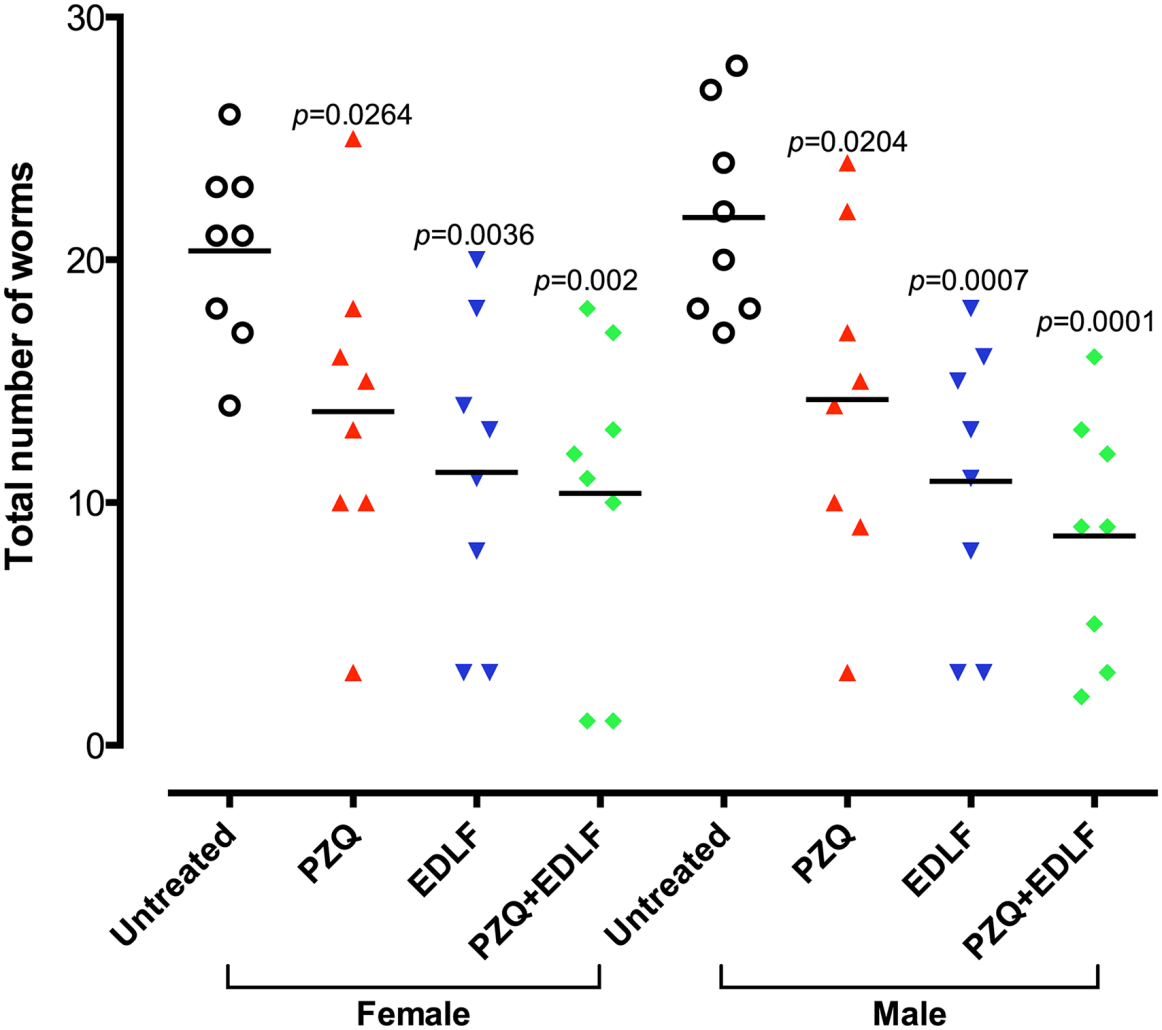
Effect on adult male and female worm burdens after PZQ, EDLF and PZQ+EDLF prophylactic treatments in mice infected with *S*. *mansoni*. Mice were treated by oral administration of 100 mg/kg/day PZQ, 45 mg/kg/day EDLF or PZQ+EDLF as prophylactic treatments for *S*. *mansoni* infection as shown in Materials and Methods. Infected untreated (*untreated*) mice were treated with vehicle. Each point represents data from an individual treated or infected untreated mouse. Horizontal bars indicate mean values. Significance (*p*) values with respect to infected untreated mice are indicated. The means ± SEM (n = 8) for each experimental condition are as follows: female (untreated: 20.38 ± 1.36; PZQ: 13.75 ± 2.29; EDLF: 11.25 ± 2.23; PZQ+EDLF: 10.38 ± 2.26) and male (untreated: 21.75 ± 1.49; PZQ: 14.25 ± 2.44; EDLF: 10.88 ± 2.03; PZQ+EDLF: 8.62 ± 1.76).

In order to study the efficacy of EDLF in a prophylactic setting for schistosomiasis, we treated four different cohorts of CD1 mice with PZQ (100 mg/kg/day), EDLF (45 mg/kg/day), PZQ+EDLF and only vehicle (infected untreated control group), since three days before until eight days after being infected with *S*. *mansoni* cercariae as indicated in Materials and Methods and Fig 1. Both PZQ and EDLF were orally administered, and live adult worm count from hepatic and portomesenteric veins as well as the size of hepatic granuloma, and the total number of eggs found in liver was determined after the eighth week of infection. All treatments reduced significantly the number of live worms as compared to infected untreated mice, with the groups treated with EDLF and PZQ+EDLF showing the highest decrease in worm count, even higher than that obtained by using PZQ alone (Fig 3).

**Fig 4:**
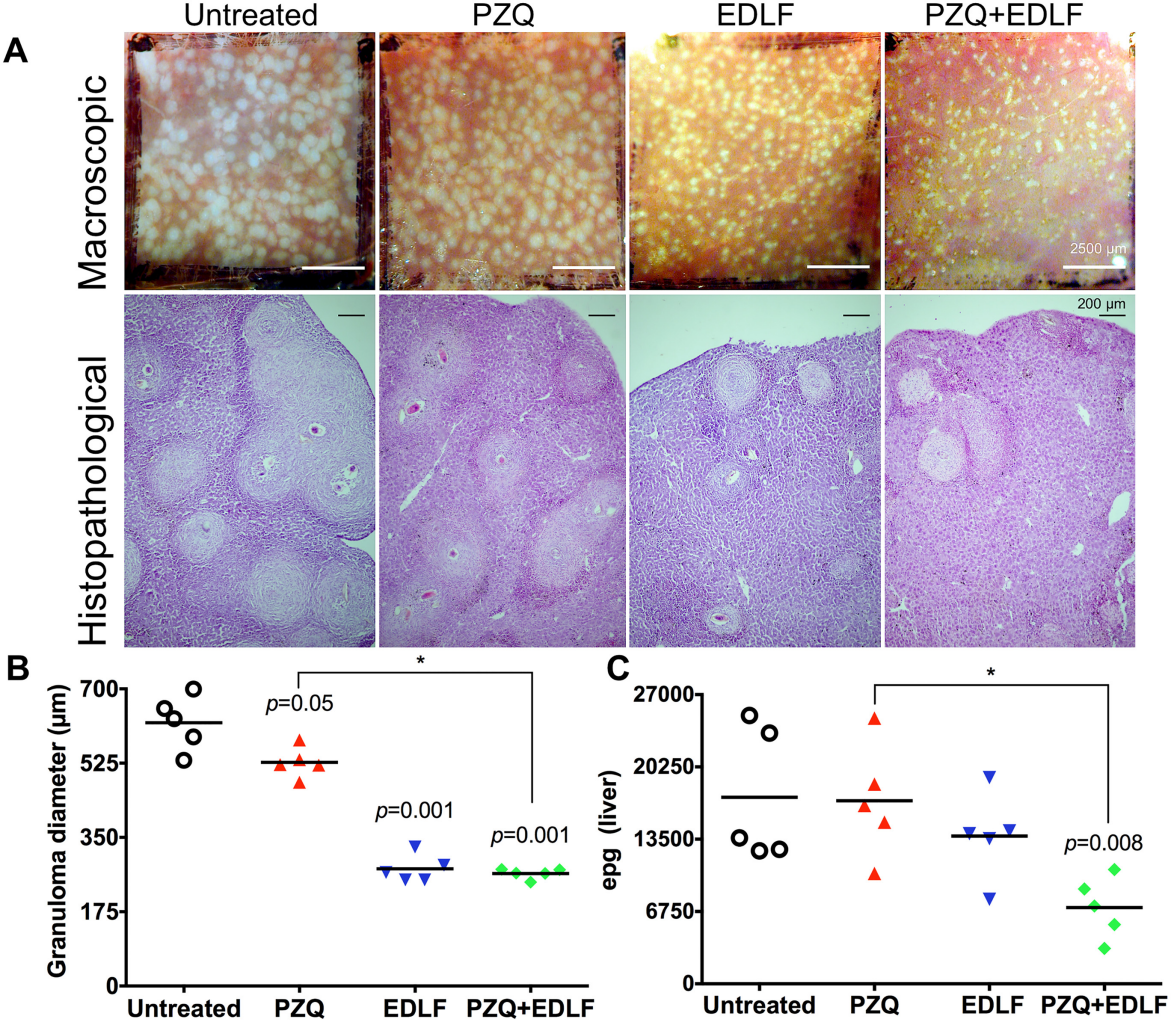
Effects on granuloma size and parasite egg burdens in liver after prophylactic treatment of *S*. *mansoni*-infected mice with PZQ, EDLF and PZQ+EDLF treatments. (A) Representative hepatic granulomas of 8-week-infected drug-untreated and drug-treated mice. Photographs were taken at 10 x. (B) Granuloma diameter. The average values of the diameters of 25 granulomas measured in liver sections from 5 infected mice per group (5 granulomas per mouse) are shown. Each point represents the value for an individual mouse. Significance (*p*) values with respect to infected untreated mice are indicated. Statistical significance between the PZQ and PZQ+EDLF groups is also included. (*) *p*<0.05. The means ± SEM (n = 5) for each experimental condition are as follows: untreated: 620.4 ± 28.70; PZQ: 526.9 ± 15.95; EDLF: 276 ± 14.41; PZQ+EDLF: 264.9 ± 5.41. (C) Parasite egg burden in liver. Infected mice were treated with 100 mg/kg PZQ, 45 mg/kg EDLF, or PZQ+EDLF. Infected untreated mice were run in parallel. Compounds were administered orally as described in Materials and Methods, and the number of eggs in liver was determined as eggs per gram (epg). Each point represents data from an individual treated- or infected untreated mouse. Horizontal bars indicate average values. Significance (*p*) values with respect to infected untreated mice are shown. Statistical significance between the PZQ and PZQ+EDLF groups is also included. (*) *p*<0.05. The means ± SEM (n = 5) for each experimental condition are as follows: untreated: 17411 ± 2805; PZQ: 17094 ± 2368; EDLF: 13796 ± 1804; PZQ+EDLF: 7126 ± 1279).

Interestingly, following both macroscopic and microscopic histopathological examination, we observed a significant reduction in hepatic granuloma size in mice treated with PZQ, EDLF and PZQ+EDLF as compared to infected untreated mice, EDLF and PZQ+EDLF being the most efficient treatments in reducing granulomatous inflammation (Fig 4A and 4B).

Because granuloma forms around the eggs we next determined the amount of eggs in liver. Intriguingly, despite the above reduction in adult worm count and hepatic granuloma size, we did not detect any statistical difference in the amount of parasite eggs in liver between the infected untreated control group and experimental groups receiving either PZQ or EDLF alone (Fig 4C). Because PZQ kills preferentially schistosoma parasites at adult stages, but is not active against immature worms [59, 60], it is interesting to note a significant reduction in the number of eggs in liver when mice were treated with PZQ+EDLF as compared with the infected untreated group. The PZQ+EDLF combination treatment induced a statistically significant (*p*<0.05) decrease in both granuloma diameter and parasite egg burden in liver as compared to PZQ-treated mice (Fig 4B and 4C). To examine the effect of drug administration on the hepatomegaly caused by *S*. *mansoni* infection, liver was excised from dissected mice after treatment, weighed and the relative liver weight in relation to body weight was calculated. As shown in S2 Fig, there was a significant decrease in the relative liver weight in PZQ+EDLF-treated mice as compared to infected untreated mice (*p*<0.05), thus suggesting that the combined PZQ+EDLF treatment alleviates hepatomegaly. Similar results were obtained when the amount of parasite eggs in intestine was measured (S3 Fig). The combined PZQ+EDLF treatment also induced a statistically significant decrease in the parasite egg burden in intestine as compared to infected untreated mice (*p*<0.01) and PZQ-treated group (*p*<0.001) (S3 Fig).

### Effect of PZQ+EDLF treatment on serum cytokine levels

**Fig 5:**
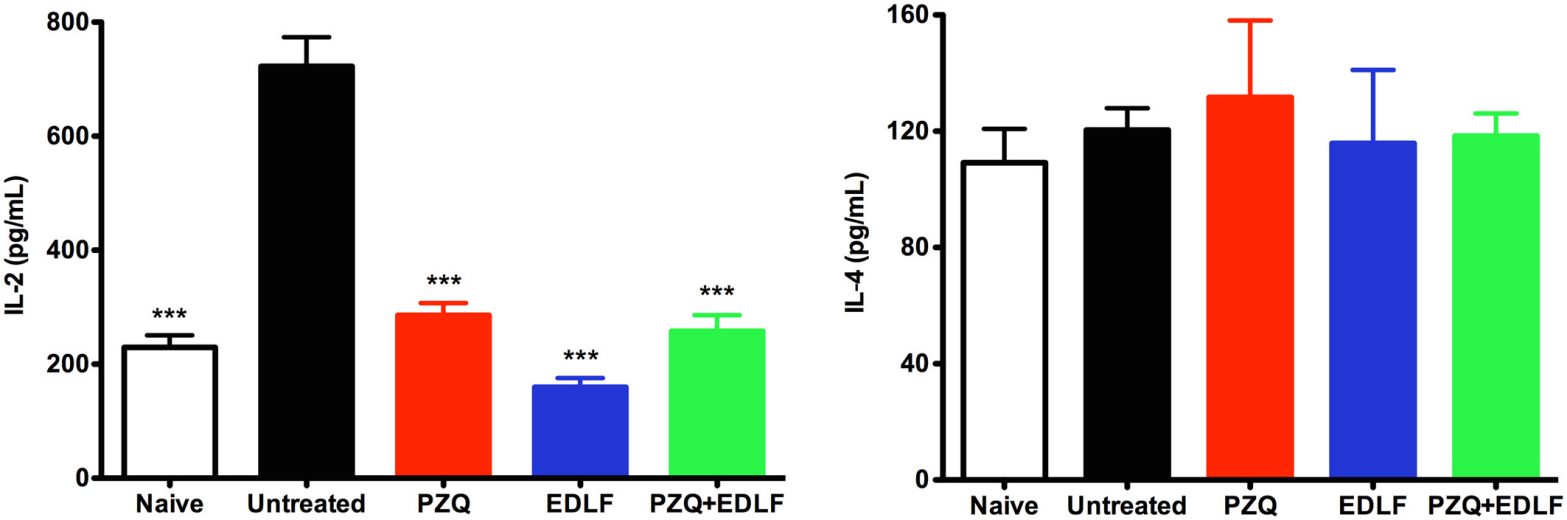
IL-2 and IL-4 plasma levels in drug treated- and untreated-mice. The plasma levels of the indicated cytokines were determined in uninfected-untreated naive mice and the distinct untreated and treated infected mice, namely infected untreated (*untreated*), PZQ, EDLF and PZQ+EDLF as shown in Materials and Methods. Samples were taken at week 3 p.i. Data are shown as means ± SEM of eight mice. Asterisks represent statistical significance with respect to the infected untreated group. (***) *p*<0.001.

**Fig 6:**
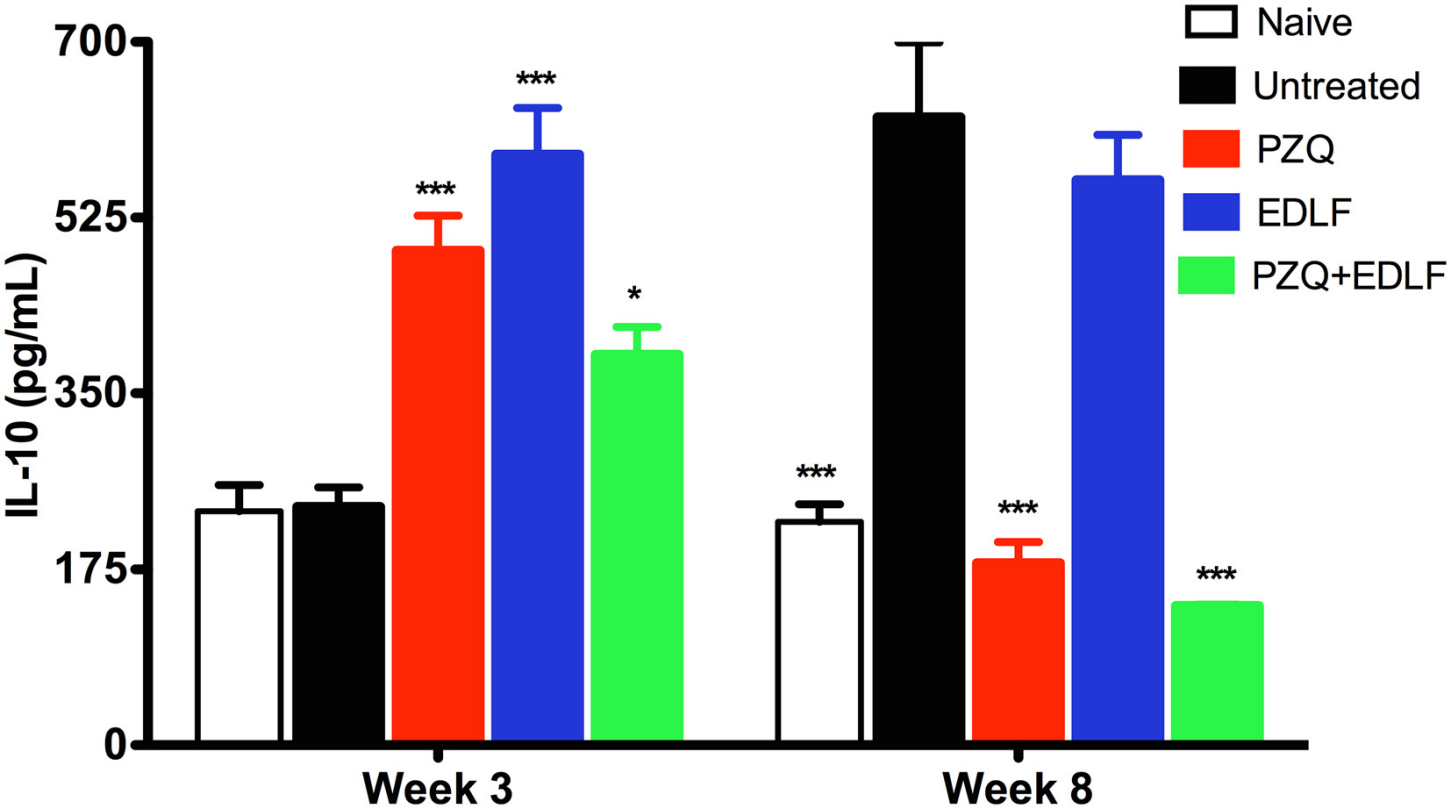
IL-10 plasma level in drug treated- and untreated-mice. The plasma levels of IL-10 were determined in uninfected-untreated naive mice and the distinct untreated and treated infected mice, namely infected untreated (*untreated*), PZQ, EDLF and PZQ+EDLF as shown in Materials and Methods. Samples were taken at weeks 3 and 8 p.i. Data are shown as means ± SEM of eight mice. Asterisks represent statistical significance with respect to the infected untreated group. (*) *p*<0.05; (***) *p*<0.001.

In order to explore the effect of EDLF-containing treatments on cellular immune response, we used a flow cytometry-based methodology to measure the levels of several cytokines in the sera of mice at 3 and 8 weeks p.i. At early stages of infection (3^rd^ week p.i.), the distinct PZQ, EDLF or PZQ+EDLF treatments drastically inhibited the infection-induced increase in IL-2 production, as a typical Th1 cytokine, whereas the Th2 cytokine IL-4 level was not affected (Fig 5). Interestingly, the above three treatments induced an increase in the level of IL-10 at week 3 p.i. (Fig 6), thus suggesting the triggering of an anti-inflammatory action as IL-10 inhibits production of pro-inflammatory cytokines [61, 62]. Because at this early stage of infection (week 3 p.i.) the IL-4 and IL-10 levels were not affected by the infection (Figs 5 and 6), these data indicated that the Th2 and Treg responses were not elicited by the parasite at this infection period, and thereby the actions detected on the IL-10 level following the above three treatments suggested a direct interaction of PZQ and EDLF with the corresponding T cell subsets. Then, after eight weeks p.i. the data on IL-10 levels differed greatly from those obtained at early stages of infection (Fig 6). As infection progresses to late stages (week 8 p.i.), infected untreated mice showed elevated levels of IL-10 in plasma (Fig 6), indicative of Treg and Th2 responses. However, treatments with PZQ and above all PZQ+EDLF led to a drastic reduction in the level of IL-10, reaching a level that was even lower than that detected in naive mice (Fig 6). Because mice were not treated any longer since the day 9 of infection, it is expected that they were free of PZQ and EDLF by the eighth week p.i., and therefore the changes in cytokine production could be due to an immunological reaction to either the surviving or dead parasites at their different developmental stages. Because IL-10 blocks the development of resistance to re-infection with *S*. *mansoni* [63], the inhibition of IL-10 production in the combined PZQ+EDLF treatment at late stages of infection, together with its drastic inhibitory action on granuloma formation and egg count, suggests that this combination treatment could be of particular interest for a prophylactic use against schistosomiasis.

**Fig 7:**
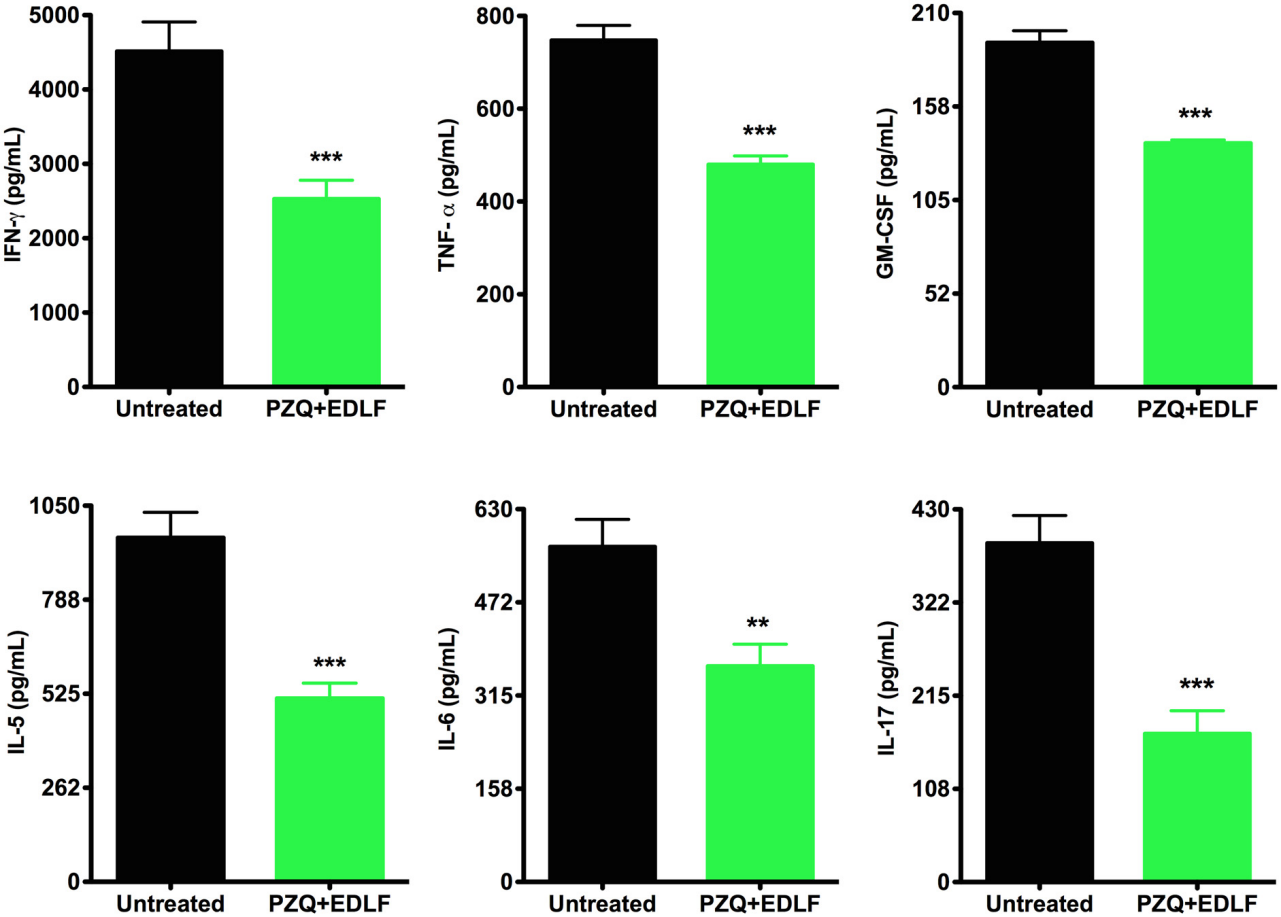
IFN-γ, TNF-α, GM-CSF, IL-5, IL-6 and IL-17 plasma levels in PZQ+EDLF-treated- and untreated infected mice. The plasma levels of the indicated cytokines were determined in infected untreated (*untreated*) and PZQ+EDLF-treated infected mice as shown in Materials and Methods. Samples were taken at week 8 p.i. Data are shown as means ± SEM of eight mice. Asterisks represent statistical significance with respect to the infected untreated group. (**) *p*<0.01; (***) *p*<0.001.

Interestingly, the levels of a number of Th1 (IFN-γ, TNF-α, GM-CSF) and Th2 (IL-5, IL-6) cytokines were significantly reduced following the PZQ+EDLF combined treatment as compared to infected untreated mice at the late stage of infection (Fig 7). Furthermore, the combined treatment of PZQ+EDLF also dramatically decreased the level of the IL-17 at the late stage of infection (Fig 7), suggesting that the pro-inflammatory Th17 response, which plays a major role in hepatic granulomatous inflammation against parasite eggs [64], was largely diminished. In addition, a dramatic reduction in the plasma level of IL-17 in the PZQ+EDLF group was also detected at week 3 p.i. (726 ± 78 *vs*. 161 ± 32 pg/mL (n = 8), *p*<0.001, between infected untreated mice and PZQ+EDLF-treated infected mice, respectively).

### Effects of EDLF-containing treatments on granulocyte count

It is well known the participation of leukocytes in inflammatory processes associated with parasitic diseases and also in the clearance of the disease, mainly due to granulocytes, which comprise neutrophils, eosinophils and basophils [65, 66]. The immune response in hepatic and intestinal tissues switches the expansion of Th2-associated myeloid cells, including eosinophils and basophils. In this regard, eosinophils have been found to constitute the majority of cells (~51%) within the hepatic granuloma in *S*. *mansoni*-infected mice [67].

**Fig 8:**
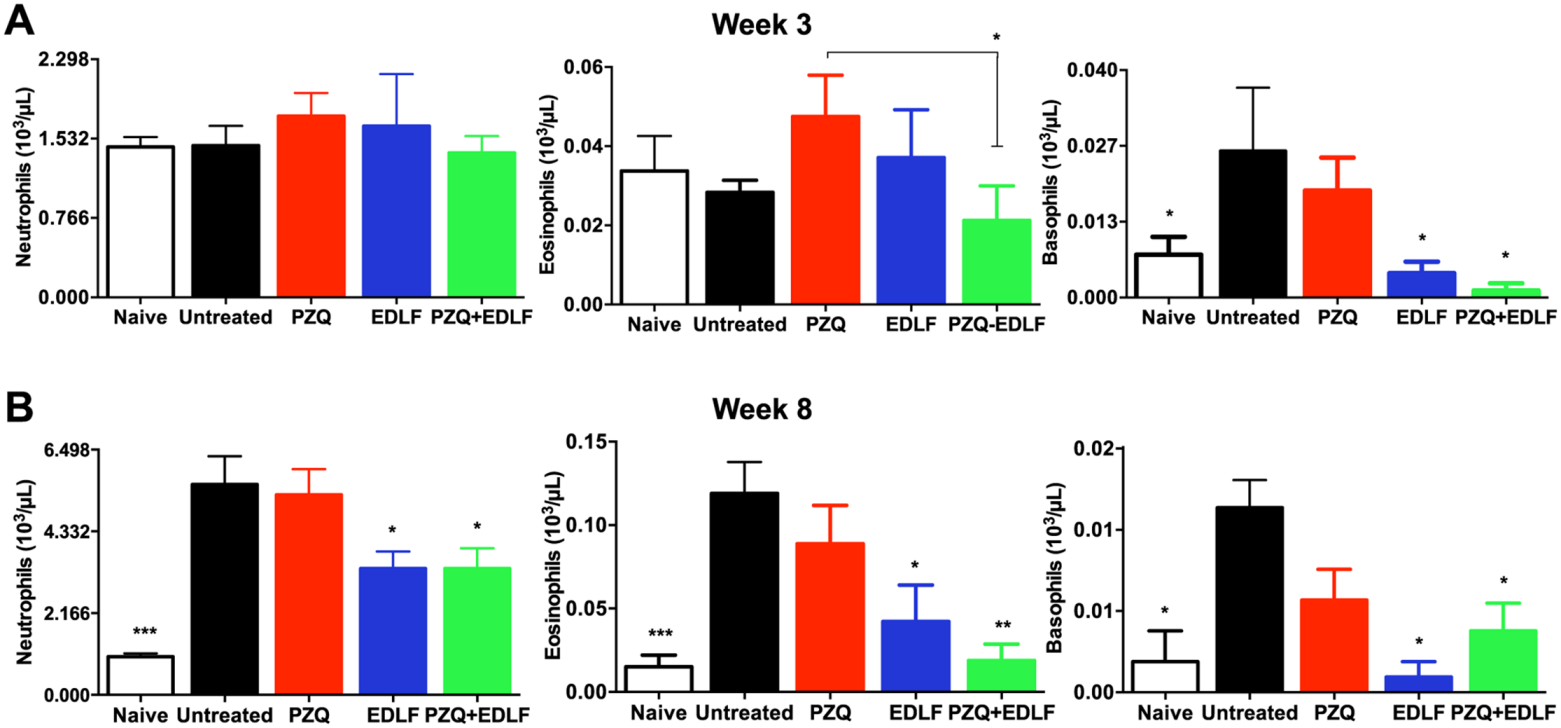
Granulocyte analysis in drug treated- and untreated-mice. Blood samples were analyzed for neutrophil, eosinophil and basophil counts in uninfected-untreated naive mice, infected untreated (*untreated*) mice and infected mice treated with PZQ, EDLF or PZQ+EDLF. Samples were taken at weeks 3 (A) and 8 (B) p.i. Data are shown as means ± SEM of eight mice. Asterisks represent statistical significance with respect to the infected untreated group. (*) *p*<0.05; (**) *p*<0.01; (***) *p*<0.001.

Here, we examined the levels of the above leukocyte types in peripheral blood in each experimental group at 3 and 8 weeks p.i. At week 3 p.i. we detected a significant reduction in the level of eosinophils in the group of mice treated with PZQ+EDLF when compared to the PZQ-treated group (Fig 8A), as well as a significant reduction in the levels of basophils in the mice treated with EDLF and PZQ+EDLF compared to the infected untreated group (Fig 8A). In the late stages of infection (week 8 p.i.), a significant increase in the number of neutrophils, eosinophils and basophils was found in the infected untreated mice compared to the naive non-infected animals (Fig 8B). Interestingly, we found a significant reduction in the levels of neutrophils, eosinophils and basophils in the groups of mice treated with EDLF and PZQ+EDLF as compared to the infected untreated group (Fig 8B). However, no significant changes were observed regarding the number of lymphocytes and monocytes in the three experimental groups as compared to infected untreated mice (S1 Table).

## Discussion

In the present study we have found that the combination of PZQ and the ether phospholipid EDLF behaves as a potent and promising prophylactic treatment for schistosomiasis. This prophylactic effect was significantly greater than those observed in the single drug treatment groups. Our results represent the first evidence that EDLF kills immature forms of *S*. *mansoni* using both *in vitro* and *in vivo* assays. Thus, we have found here that EDLF kills schistosomula, and both PZQ [59, 60] and EDLF [35] have been previously shown to be effective drugs against *S*. *mansoni* adult worms. Recently, we have shown that EDLF reduces worm burden in a murine model [35], and we report here a statistically significant decrease in worm count in an *in vivo* assay following a prophylactic treatment with EDLF or the PZQ+EDLF combination treatment that was higher than that detected following PZQ prophylactic treatment. PZQ is less active against the juvenile stages of *S*. *mansoni* than the adult schistosomes [59, 60]. The minor activity of PZQ against juvenile schistosomes is believed to be a key factor explaining the observed treatment ‘failures’ in areas highly endemic for schistosomiasis and that require frequent retreatments [32, 68]. The relative resistance of the larval stages of *S*. *mansoni* to schistosomicide drugs may result in a therapeutic failure because of the presence of migrating, drug-resistant, immature forms of the parasite [69]. On the other hand, although the existing antischistosomal drugs are highly effective against adult worms, they do not prevent against re-infection or granuloma formation [70, 71]. In this context, the present results on the killing activity of EDLF on *S*. *mansoni* schistosomula are of major importance in the development of effective schistosomicide drugs. It is worth mentioning that very recent evidence shows that EDLF elicits a selective and direct killing on soil-dwelling nematode *Caenorhabditis elegans* embryos [72]. Taken together, these data suggest that EDLF is able to kill helminths at both early and late developmental stages. The mechanism of action of EDLF on schistosomes is still unclear. In this regard, it is worth mentioning that EDLF is a proapoptotic agent in cancer cells [38, 41, 42] affecting processes at the membrane level [37, 40, 73, 74], and recent evidence suggests that EDLF promotes an apoptosis-like process in *Leishmania* spp. and *S*. *mansoni* adult worms [35, 75]. APLs, including miltefosine and EDLF, have been recently shown to exert schistosomicide activity on various species and strains of schistosomes [33–35, 76, 77], by interfering with membrane stability and structural integrity of worms’ tegument, and resulting in marked alterations of the digestive tract and the reproductive system of the worms. In addition, it has been very recently shown that Akt inhibition induces profound alterations in *S*. *mansoni* adult worm pairing and egg laying as well as affects the viability of schistosomula larvae [78], and EDLF also inhibits Akt signaling in cancer cells [79].

Interestingly, the herein reported treatments containing EDLF promoted a significant and high decrease in granuloma formation as well as in the immune response that underlies granuloma development. EDLF at the early stages p.i. drastically inhibited the infection-induced IL-2 generation, while upregulated IL-10, which inhibits Th1 inflammatory response. These data agree with recent data showing an anti-inflammatory effect of EDLF on different animal models for distinct diseases [45–47]. Elevated serum levels of IL-10 have also been observed in PZQ-treated humans, which paralleled an elevated anti-worm Th2 response [80]. Surprisingly, the combination of PZQ+EDLF induced a significant increase in the IL-10 level at early p.i. times, whereas this cytokine level was dramatically inhibited at late p.i. stages. This is of major importance as studies of human schistosomiasis indicate the importance of IL-10 in regulating morbidity [17], and IL-10 has been shown to inhibit the development of protective immunity to secondary schistosome infection [63]. Thus, blockade of IL-10 combined with PZQ treatment raised protective immunity against re-infection with *S*. *mansoni* [63].

Interestingly, we also found here that the combined treatment of EDLF and PZQ led to a marked inhibition in the Th1, Th2 and Th17 responses at late *S*.*mansoni* p.i. times. Prolonged Th2 [81] and Th17 [82] responses contribute to the development of hepatic granulomatous inflammation and hepatic fibrosis, and thereby the drastic reduction in the level of Th2 and Th17 cytokines in mice treated with PZQ+EDLF reported here could explain in part the significant reduction in granuloma formation.

Th2 immunity involves the rapid activation and engagement of cells of both the innate (eosinophils and basophils) and adaptive (CD4^+^ T cells committed to the Th2 pathway) immune systems [83], and constitutes a crucial factor in the generation of granuloma. Eosinophils play a role in the killing of schistosomula at about four weeks of infection [84] and constitute the major cell population in hepatic granulomas (~51%) [66, 67]. Basophils are found in very small numbers in the circulation (0.01 to 0.3% of total leukocytes), and following activation they secrete a number of mediators including histamine, leukotrienes, proteoglycans and proteolytic enzymes, as well as several cytokines, playing a major role in inflammation and modulating the number of eosinophils and neutrophils present at the inflammatory site [85, 86]. Basophils have been thought to migrate into inflamed tissues after the Th2 cytokine-dependent response is established, and they are associated with chronic allergic inflammation and helminth infections [85, 87]. Recent studies have demonstrated that MHC class II^+^ murine basophils migrate to the draining lymph nodes following exposure to *S*. *mansoni* eggs [88], and depletion of basophils resulted in a concomitant downregulation of egg granuloma formation at 7 weeks p.i. [87]. In IL-17-associated pathogenicity of schistosomiasis, Th17 response favors neutrophil accumulation and degranulation, thus exacerbating egg-induced tissue damage [31, 89, 90]. The results reported here show a marked decrease at late p.i. times in the number of neutrophils, eosinophils and basophils in the blood of *S*. *mansoni*-infected mice, treated with EDLF or PZQ+EDLF, further supporting an anti-inflammatory effect of EDLF-containing therapies, and thus ameliorating hepatic granulomatous inflammation and liver damage. However, no significant changes were detected in the total number of blood lymphocytes and monocytes following pretreatment with PZQ, EDLF or PZQ+EDLF when compared to infected untreated mice, showing figures similar to those previously reported [91]. On these grounds, our data indicate that the inclusion of EDLF in the prophylactic regimen leads to a dramatic change in the immune response elicited following *S*. *mansoni* infection. However, EDLF does not act by indiscriminately eliminating cells in secondary lymphoid organs that are crucial for triggering antigen- specific immunity [47]. Moreover, we have also shown here that PZQ+EDLF combination treatment significantly reduced the amount of eggs in both liver and intestine. Taken together, our data suggest that the inclusion of EDLF in combination therapy regimens improves schistosomiasis prophylaxis.

Because in our present study mice were treated with PZQ and EDLF since three days before until eight days after infection, and the different experimental determinations were performed 3 and 8 weeks p.i., an important factor to take into account is the pharmacokinetic parameters of both drugs. Whereas the elimination half-life of PZQ is between 1–3 h [92, 93], the APL EDLF shows a much slower elimination rate (half-life of elimination, 30.4 ± 26.8 h). EDLF has also a high distribution to tissues, being highly distributed extravascularly, and with a rapid distribution to organs that are highly irrigated, including liver, where it shows a low hepatic clearance value [94]. The APL miltefosine shows an extremely slow elimination, as assessed by the long elimination half-lives estimated from a two-compartment pharmacokinetic model, with a primary elimination half-life of 7.05 days and a terminal half-life of 30.9 days [43, 95]. Miltefosine is eliminated from the body at a very slow rate and is still detectable in human plasma samples taken 5 to 6 months after the end of treatment [95]. Thus, APLs seem to be characterized by their long residence times in the body. This long elimination half-life could be of importance for the prophylactic action of EDLF and EDLF-containing regimens.

Combination therapy, ideally among drugs with unrelated mechanisms of action and targeting the different developmental stages of schistosomes in the human host, could be pursued as an area for future research [96, 97]. Here, we have found that the combination of PZQ and EDLF leads to a prophylactic treatment that promotes the killing of mature and immature forms of *S*. *mansoni*, as well as a drastic reduction in the immune response at late p.i. times that could lead to a significant decrease in granuloma formation and liver pathology.

In conclusion, the results of this study demonstrate that the PZQ+EDLF combination prophylactic treatment described here is able to kill immature forms of *S*. *mansoni*, and modulate immune responses of infected mice, leading to a significant reduction in parasite burden and hepatic granuloma size. In addition, it is tempting to envisage that the combination of PZQ and EDLF could be a promising therapeutic regimen not only for prophylaxis treatment, but also for combination therapy against schistosomiasis. The results reported here warrant further studies on the putative use of the alkylphospholipid EDLF together with PZQ as a promising approach for treating schistosomiasis.

## Supporting Information

S1 Fig Differential morphology and propidium iodide permeability between live and dead *S*. *mansoni* schistosomula. Image shows two schistosomula, alive (bottom) and dead (top), under light (left) and fluorescence (right) microscopy. Dead schistosomula show loss in membrane permeability leading to propidium iodide staining, as well as tegumental deformation and a granular appearance in contrast to control live parasites, by fluorescence microscopy (propidium iodide staining) and light microscopy morphology.
(TIF)

S2 Fig Effects on relative liver weight after prophylactic treatment of *S*. *mansoni*-infected mice with PZQ, EDLF or PZQ+EDLF. Infected mice were treated with 100 mg/kg PZQ, 45 mg/kg EDLF, or PZQ+EDLF. Control groups consisting of a normal untreated (*naive*) and an infected untreated (*untreated*) group, were given the same amount of the vehicle at the same time as the PZQ-, EDLF- or PRQ+EDLF-treated groups and were run in parallel. Compounds were orally administered. Relative liver weight was determined as follows: Relative liver weight = (absolute liver weight/body weight) x 100. Each point represents data from an individual drug-treated- or infected untreated-mouse. Horizontal bars indicate average values. Significance (*p*) value with respect to infected untreated mice is indicated. The means ± SEM (n = 7) for each experimental condition are as follows: naive (4.3 ± 0.07); untreated (7.95 ± 0.42); PZQ: (7.13 ± 0.13); EDLF: 6.85 ± 0.44; PZQ+EDLF: 6.19 ± 0.55).
(TIF)

S3 Fig Effects on parasite egg burden in intestine after prophylactic treatment of *S*. *mansoni*-infected mice with PZQ, EDLF or PZQ+EDLF. Infected mice were treated with 100 mg/kg PZQ, 45 mg/kg EDLF, or PZQ+EDLF. Infected untreated mice were run in parallel. Compounds were orally administered. Parasite egg burden in intestine was determined as eggs per gram (epg). Each point represents data from an individual treated- or infected untreated-mouse. Horizontal bars indicate average values. Significance (*p*) values with respect to infected untreated mice are indicated. Statistical significance between the PZQ and PZQ+EDLF groups is also included. (***) *p*<0.001. The means ± SEM (n = 5) for each experimental condition are as follows: untreated (18532 ± 1600); PZQ (22435 ± 1160); EDLF (13872 ± 3333); PZQ+EDLF (4306 ± 1465).
(TIFF)

S1 Table White blood cell, lymphocyte and monocyte count in drug-treated and untreated mice. (XLSX)

S1 Video *S*. *mansoni* schistosomula in culture. This video shows a 24-h culture of *S*. *mansoni* schistosomula prepared by repeatedly passing the cercariae through a double-ended needle connected to two syringes to remove tails.
(MOV)

S2 Video *In vitro* effect of edelfosine on *S*. *mansoni* schistosomula. These movies show morphological changes in control untreated schistosomula (live control), heat-killed schistosomula (56°C, dead control), and schistosomula treated with 20 μM edelfosine for 24 h.
(MOV)
