# Supplementary figures and images for: Inhibition of Granulomatous Inflammation and Prophylactic Treatment of Schistosomiasis with a Combination of Edelfosine and Praziquantel
Source: PLoS Negl Trop Dis. 2015 Jul 20;9(7):e0003893. doi: 10.1371/journal.pntd.0003893 (PMC4507859; doi:10.1371/journal.pntd.0003893)

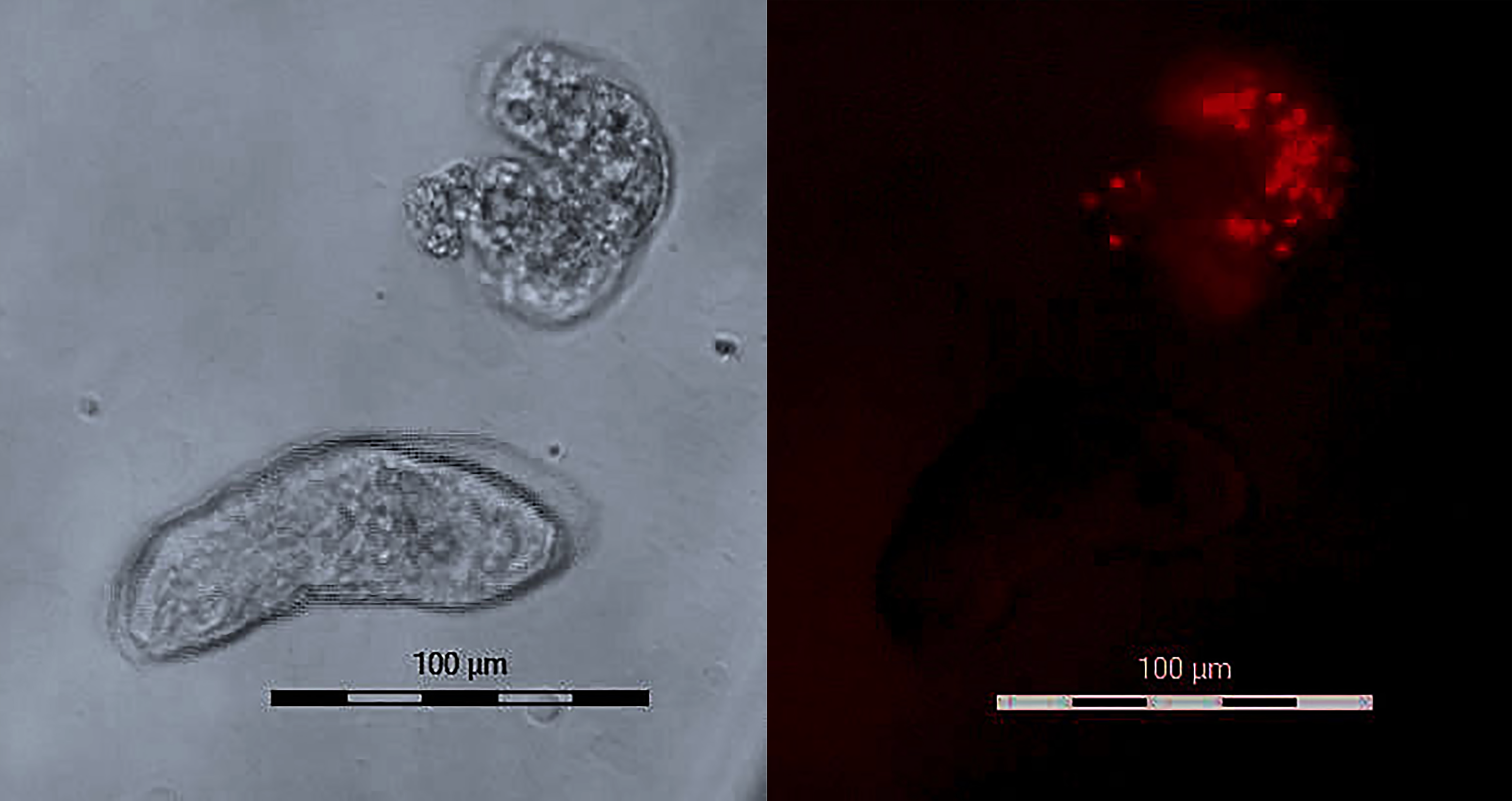

Supplement: S1 Fig — Image shows two schistosomula, alive (bottom) and dead (top), under light (left) and fluorescence (right) microscopy. Dead schistosomula show loss in membrane permeability leading to propidium iodide staining, as well as tegumental deformation and a granular appearance in contrast to control live parasites, by fluorescence microscopy (propidium iodide staining) and light microscopy morphology. (TIF) [file pntd.0003893.s001.tif]

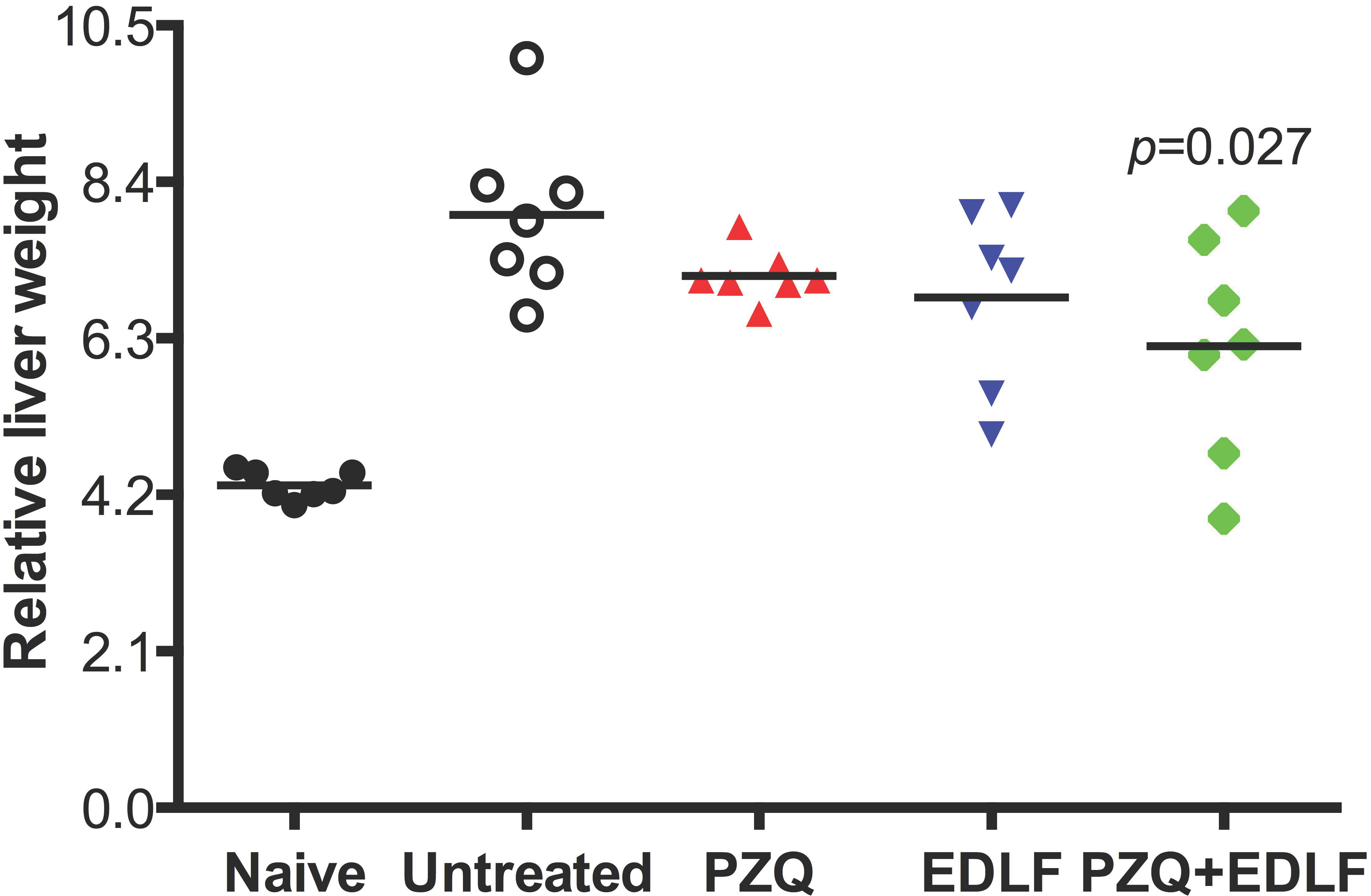

Supplement: S2 Fig — Infected mice were treated with 100 mg/kg PZQ, 45 mg/kg EDLF, or PZQ+EDLF. Control groups consisting of a normal untreated (naive) and an infected untreated (untreated) group, were given the same amount of the vehicle at the same time as the PZQ-, EDLF- or PRQ+EDLF-treated groups and were run in parallel. Compounds were orally administered. Relative liver weight was determined as follows: Relative liver weight = (absolute liver weight/body weight) x 100. Each point represents data from an individual drug-treated- or infected untreated-mouse. Horizontal bars indicate average values. Significance (p) value with respect to infected untreated mice is indicated. The means ± SEM (n = 7) for each experimental condition are as follows: naive (4.3 ± 0.07); untreated (7.95 ± 0.42); PZQ: (7.13 ± 0.13); EDLF: 6.85 ± 0.44; PZQ+EDLF: 6.19 ± 0.55). (TIF) [file pntd.0003893.s002.tif]

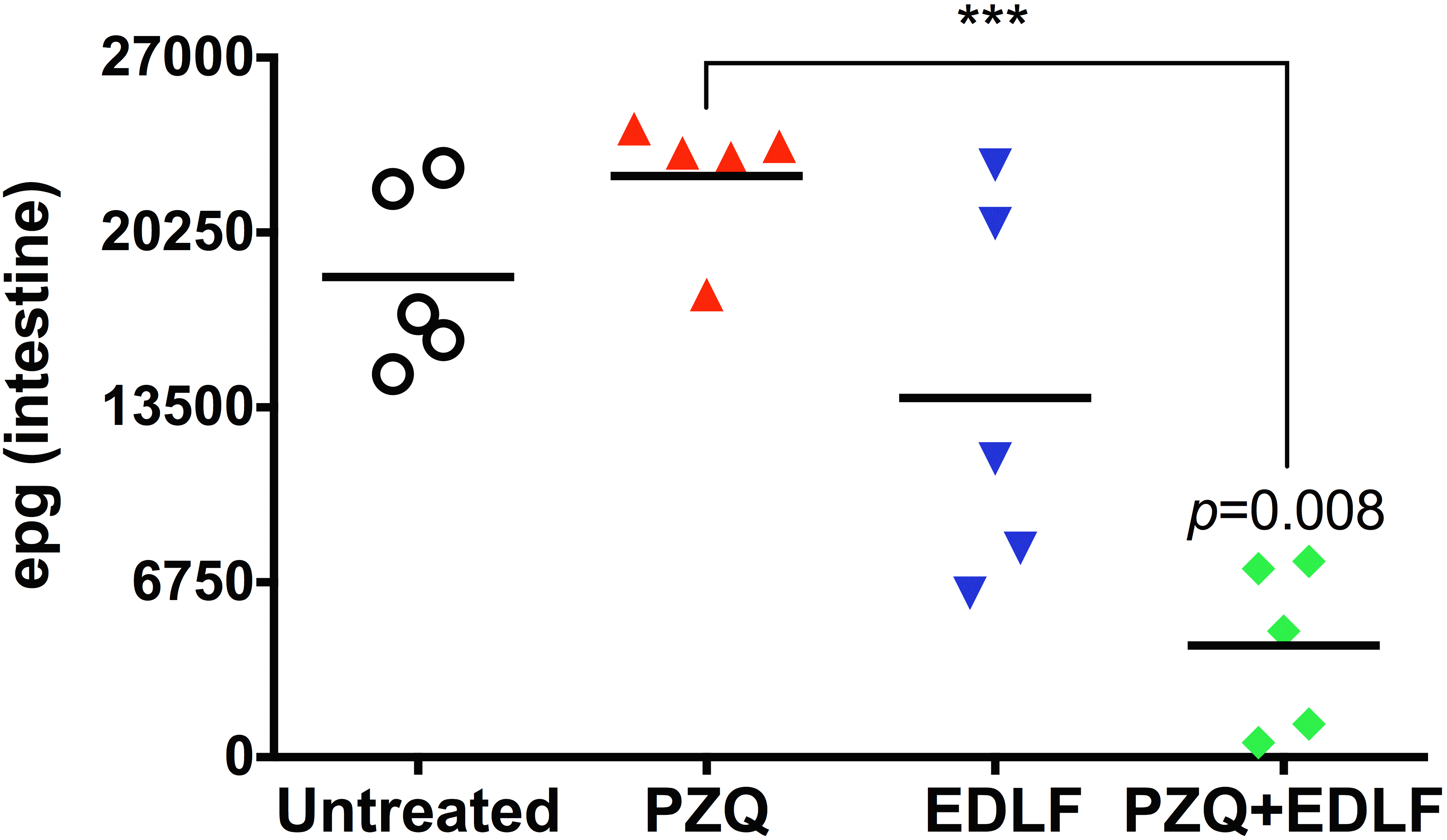

Supplement: S3 Fig — Infected mice were treated with 100 mg/kg PZQ, 45 mg/kg EDLF, or PZQ+EDLF. Infected untreated mice were run in parallel. Compounds were orally administered. Parasite egg burden in intestine was determined as eggs per gram (epg). Each point represents data from an individual treated- or infected untreated-mouse. Horizontal bars indicate average values. Significance (p) values with respect to infected untreated mice are indicated. Statistical significance between the PZQ and PZQ+EDLF groups is also included. (***) p<0.001. The means ± SEM (n = 5) for each experimental condition are as follows: untreated (18532 ± 1600); PZQ (22435 ± 1160); EDLF (13872 ± 3333); PZQ+EDLF (4306 ± 1465). (TIFF) [file pntd.0003893.s003.tiff]
